# Supplementary material for: Heterologous expression of a glycosyl hydrolase and cellular reprogramming enable Zymomonas mobilis growth on cellobiose
Source: PLoS One. 2020 Aug 14;15(8):e0226235. doi: 10.1371/journal.pone.0226235 (PMC7428164; doi:10.1371/journal.pone.0226235)
Supplement: S1 Table — (DOCX) [file pone.0226235.s001.docx]

## **S1 Table. Primers used in this study.**

| **Primer name** | **Sequence 5’ to 3’** | **Substrate** | **Comments** |
| --- | --- | --- | --- |
| 9808 | TTAATTCTAGATGTGTGAAATTGTTATCC | pIND4-spec | Vector backbone |
| 9809 | CCTTAAACGCCTGGGGTAATG |  |  |
| 9810 | tttcacacatctagaattaaTTAACTTTAAGAAGGAGATATACATATGAAAGATGATTTCC | gDNA (*C. japonicus*) | *cel3A* |
| 9811 | cccaggcgtttaaggTTAGGGGCAGGCGACGTC |  |  |
| 10244_pIND4-rev | gttagttctccttcttatgtctaatgatcgttCTCCCATGGTTAATTTCTCCTC | pIND4-spec | Backbone |
| 10245_pIND4-for | CCTTAAACGCCTGGGGTAATG |  |  |
| 10246_rbs.5-CC0968-for | gaaattaaccatgggag**aacgatcattagacataagaaggagaactaac**ATGATTTCGACGACCTTGCGGAG | gDNA (*C. crescentus*) | CC_0968 |
| 10247_rbs.5-CC0968-rev | cccaggcgtttaaggCTACTTCGCCGCCGGGGA |  |  |
| #10246_rbs.5-CC0968-for | gaaattaaccatgggag**aacgatcattagacataagaaggagaactaac**ATGATTTCGACGACCTTGCGGAG | gDNA (*C. crescentus*) | CC_0968 |
| 10252 rbs.5-CC0968-rev | tctgcgtttgtttccgtCTACTTCGCCGCCGGGGA |  |  |

Sequences in the capital letters are the sequence binding to the priming site. Sequences in **bold** are the sequence designed for high translation initiation rate predicted by ribosome binding site (RBS) calculator (<https://salislab.net/software/predict_rbs_calculator>).
