# Supplementary material for: Heterologous expression of a glycosyl hydrolase and cellular reprogramming enable Zymomonas mobilis growth on cellobiose
Source: PLoS One. 2020 Aug 14;15(8):e0226235. doi: 10.1371/journal.pone.0226235 (PMC7428164; doi:10.1371/journal.pone.0226235)
Supplement: S2 Table — (DOCX) [file pone.0226235.s002.docx]

## **S2 Table. Plasmids and strains used in this study.**

| **Plasmid** | **Backbone** | **Insert (gene)** | **Gene source** | **Reference** | |  |
| --- | --- | --- | --- | --- | --- | --- |
| pVector | pIND4-spec | None |  | This study | |  |
| pCel3A | pIND4-spec | *cel3A* | *Cellvibrio japonicus* | This study | |  |
| pGH3 | pIND4-spec | *CC_0968* (β*-*glucosidase*)* | *Caulobacter crescentus* | This study | |  |
| pGH3T | pIND4-spec | *CC_0968* (β*-*glucosidase*)–CC_0970* (TonB receptor) | *Caulobacter crescentus* | This study | |  |
| **Strain** | | **Description** | | |  | |
| *Z. mobilis* GH3 | | *Z. mobilis* ZM4 transformed with pGH3 | | | This study | |
| *Z. mobilis* control | | *Z. mobilis* ZM4 transformed with pVector | | | This study | |
