# Supplementary material for: Heterologous expression of a glycosyl hydrolase and cellular reprogramming enable Zymomonas mobilis growth on cellobiose
Source: PLoS One. 2020 Aug 14;15(8):e0226235. doi: 10.1371/journal.pone.0226235 (PMC7428164; doi:10.1371/journal.pone.0226235)
Supplement: S3 Table — (DOCX) [file pone.0226235.s003.docx]

## **S3 Table. Localization prediction of glycosyl hydrolase used in this study.**

| **Enzyme** | **Class** | **Source** | **Localization prediction** | |
| --- | --- | --- | --- | --- |
|  |  |  | **LipoP 1.0^*^** | **PSORTb^#^** |
| Cel3A | GH-3 | *Cellvibrio japonicus* | Inner membrane | Periplasmic |
| CC_0968 | GH-3 | *Caulobacter crescentus* | Periplasmic | Multiple locations |

^*^ See Reference 21.

^#^ See Reference 22.
