# Supplementary material for: Heterologous expression of a glycosyl hydrolase and cellular reprogramming enable Zymomonas mobilis growth on cellobiose
Source: PLoS One. 2020 Aug 14;15(8):e0226235. doi: 10.1371/journal.pone.0226235 (PMC7428164; doi:10.1371/journal.pone.0226235)
Supplement: S4 Table — (DOCX) [file pone.0226235.s004.docx]

## **S4 Table. Enriched gene ontology (GO) terms in both upregulated and downregulated intracellular and extracellular fractions of cellobiose- and sucrose-adapted *Z. mobilis* ZM4+pGH3 strain relative to glucose grown cells.**

**Cellobiose (Intracellular)**

| **GO term** | **No. of proteins** | **Enrichment** | **FDR adjusted Q value** |
| --- | --- | --- | --- |
| Oxidoreductase Activity | 28 | 9.4725 | 0.0000024 |
| Periplasmic Space | 8 | 11.8407 | 0.056310 |
| Cell Outer Membrane | 18 | 4.5210 | 0.11139 |
| Transport | 20 | 3.0447 | 0.2848 |

**Sucrose (Intracellular)**

| **GO term** | **No. of proteins** | **Enrichment** | **FDR adjusted Q value** |
| --- | --- | --- | --- |
| Oxidoreductase Activity | 28 | 6.7923 | 0.00854 |
| Periplasmic Space | 8 | 2.2261 | 0.076474 |
| Cell Outer Membrane | 18 | 4.0754 | 0.12568 |

**Cellobiose (Extracellular)**

| **GO term** | **No. of proteins** | **Enrichment** | **FDR adjusted Q value** |
| --- | --- | --- | --- |
| Integral Component of Membrane | 121 | 2.9218 | 0.00000175 |
| Transporter Activity | 17 | 7.0495 | 0.01222 |
| Oxidoreductase Activity | 26 | 3.6188 | 0.06385 |
| Protein Secretion | 4 | 14.8040 | 0.34677 |

**Sucrose (Extracellular)**

| **GO term** | **No. of proteins** | **Enrichment** | **FDR adjusted Q value** |
| --- | --- | --- | --- |
| Oxidoreductase Activity | 26 | 2.7845 | 0.27310 |
| Transferase Activity | 25 | 2.3867 | 0.31138 |
| Translation | 47 | 0.1350 | 0.2543 |
| Extracellular Region | 5 | 11.3370 | 0.2543 |

FDR, false discovery rate.
