# Supplementary figures and images for: Heterologous expression of a glycosyl hydrolase and cellular reprogramming enable Zymomonas mobilis growth on cellobiose
Source: PLoS One. 2020 Aug 14;15(8):e0226235. doi: 10.1371/journal.pone.0226235 (PMC7428164; doi:10.1371/journal.pone.0226235)

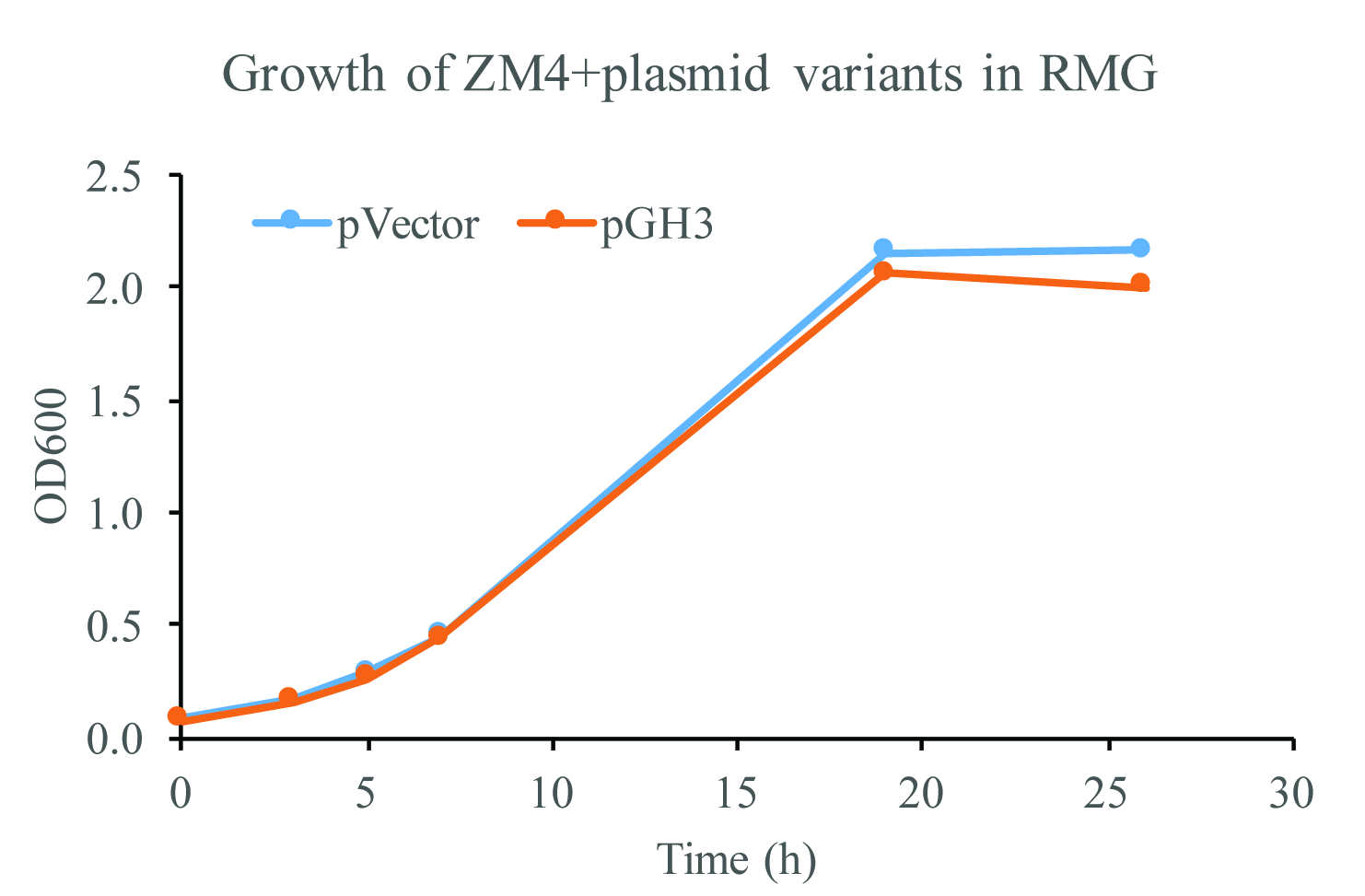

Supplement: S1 Fig — (TIF) [file pone.0226235.s005.tif]

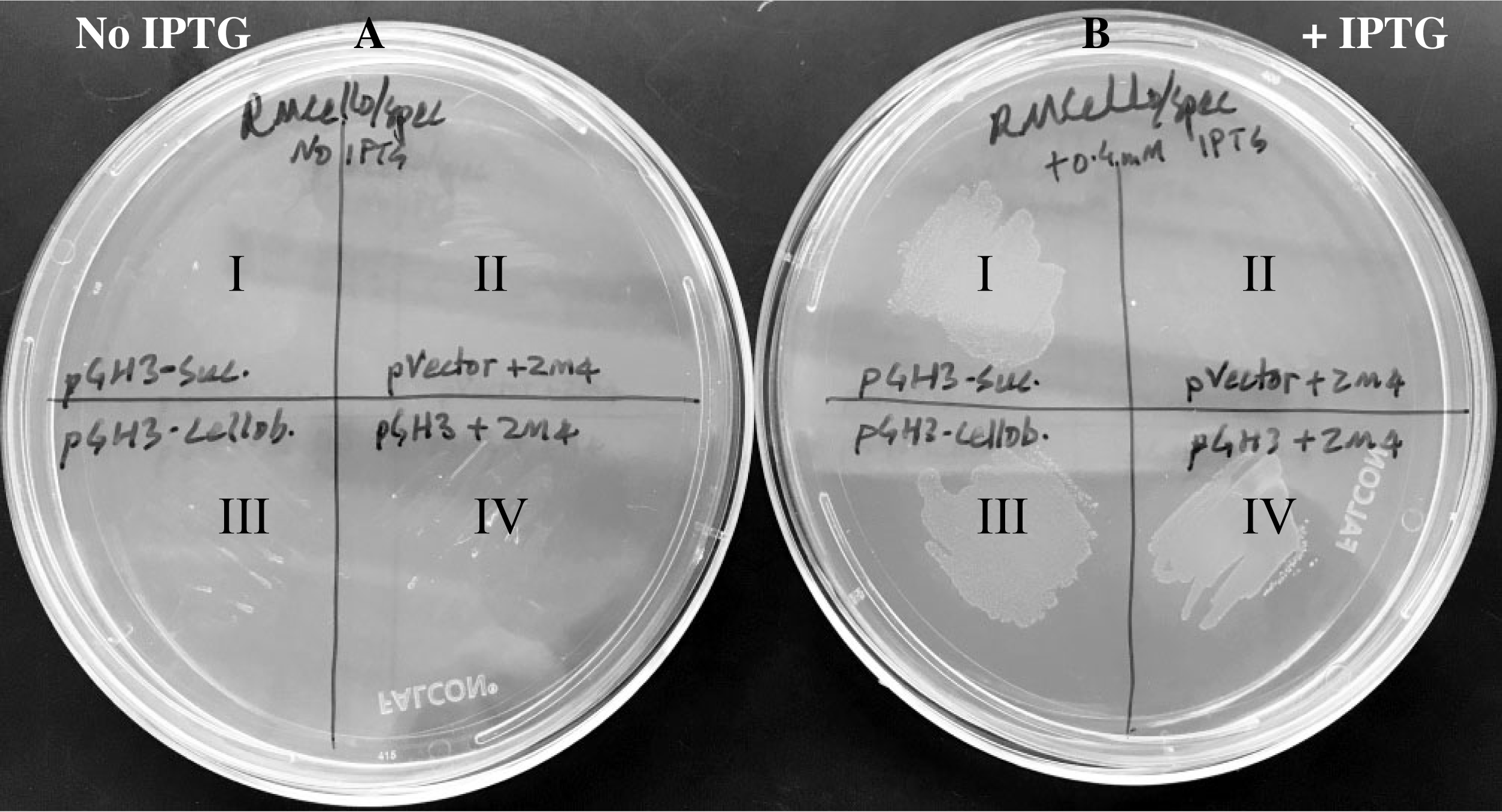

Supplement: S2 Fig — The left plate (A) is RMC with 100 μg spectinomycin/mL and no IPTG. The right plate (B) is RMC with 100 μg spectinomycin/mL and 0.4 mM IPTG. (TIF) [file pone.0226235.s006.tif]

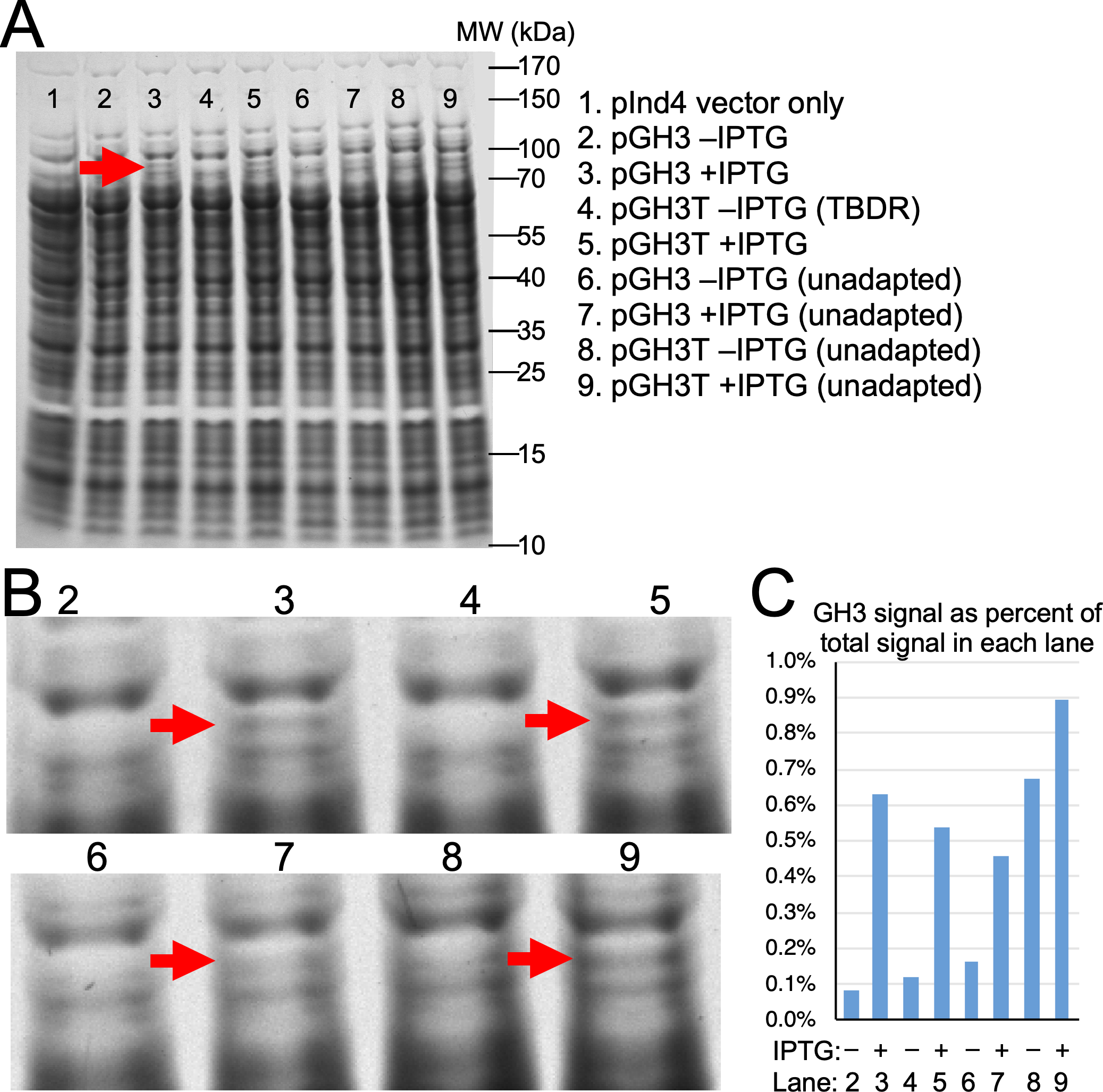

Supplement: S3 Fig — (A) SDS-PAGE showing total crude proteins. (B) Highlighted showing GH3 produced in the sample with IPTG induction. (C) GH3 signal measured as percent of total signal in each lane. (TIF) [file pone.0226235.s007.tif]

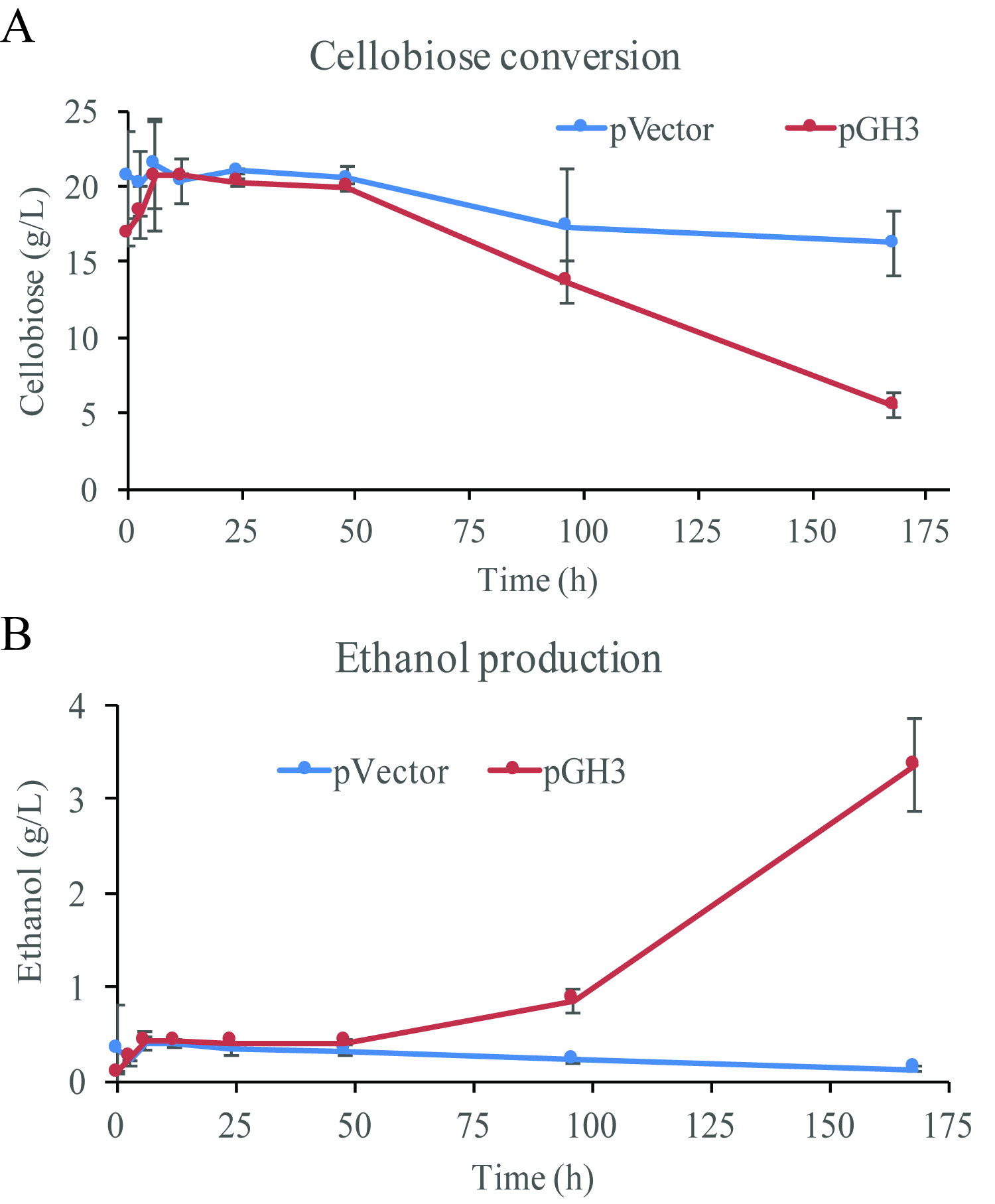

Supplement: S4 Fig — (A) Cellobiose conversion by Z. mobilis ZM4 strains containing pVector or pGH3 plasmids in RMCG medium. Cellobiose conversion was observed after 96 h only in the strain expressing glycosyl hydrolase (pGH3) but not in the control with pVector. (B) Ethanol production was observed after 96 h only in the strain expressing glycosyl hydrolase (pGH3) but not in the control with pVector. Some ethanol may have evaporated with escaping CO2 or during sampling for apparent OD600 measurement. Error bars are standard deviations of triplicate experiments. (TIF) [file pone.0226235.s008.tif]

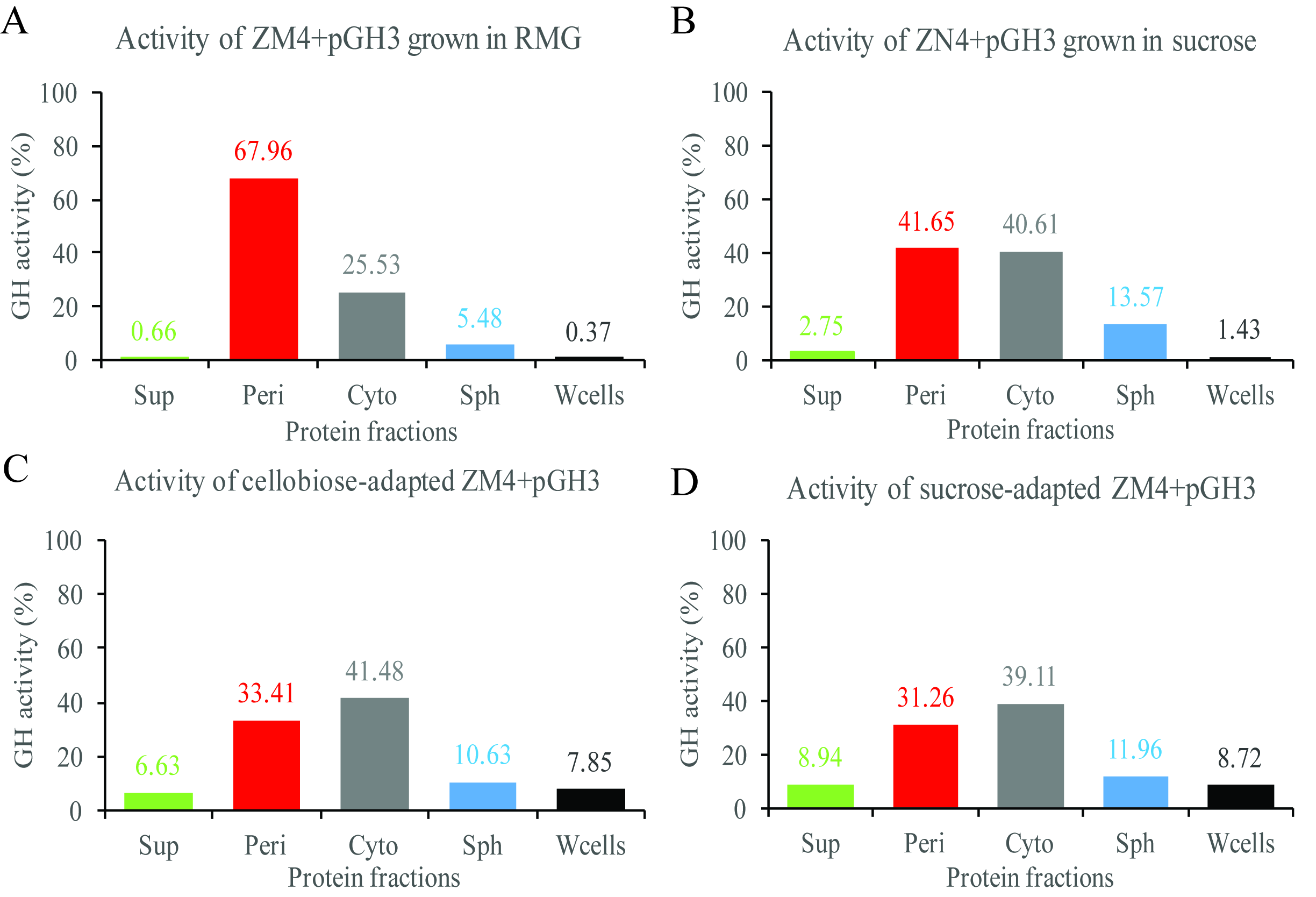

Supplement: S5 Fig — GH activity by different cellular fractions of the unadapted strain grown in RMG (A) and rich medium sucrose (B). Similarly, GH activity of different cellular fractions of cellobiose-adapted strain grown in RMCG (C), and GH activity of different cellular fractions of sucrose-adapted strain grown in RMCG (D). Abbreviations: Sup–supernatant, Peri–periplasmic fraction, Cyto–cytoplasmic fraction, Sph–spheroplast and Wcells–whole cells (cell pellets). (TIF) [file pone.0226235.s009.tif]

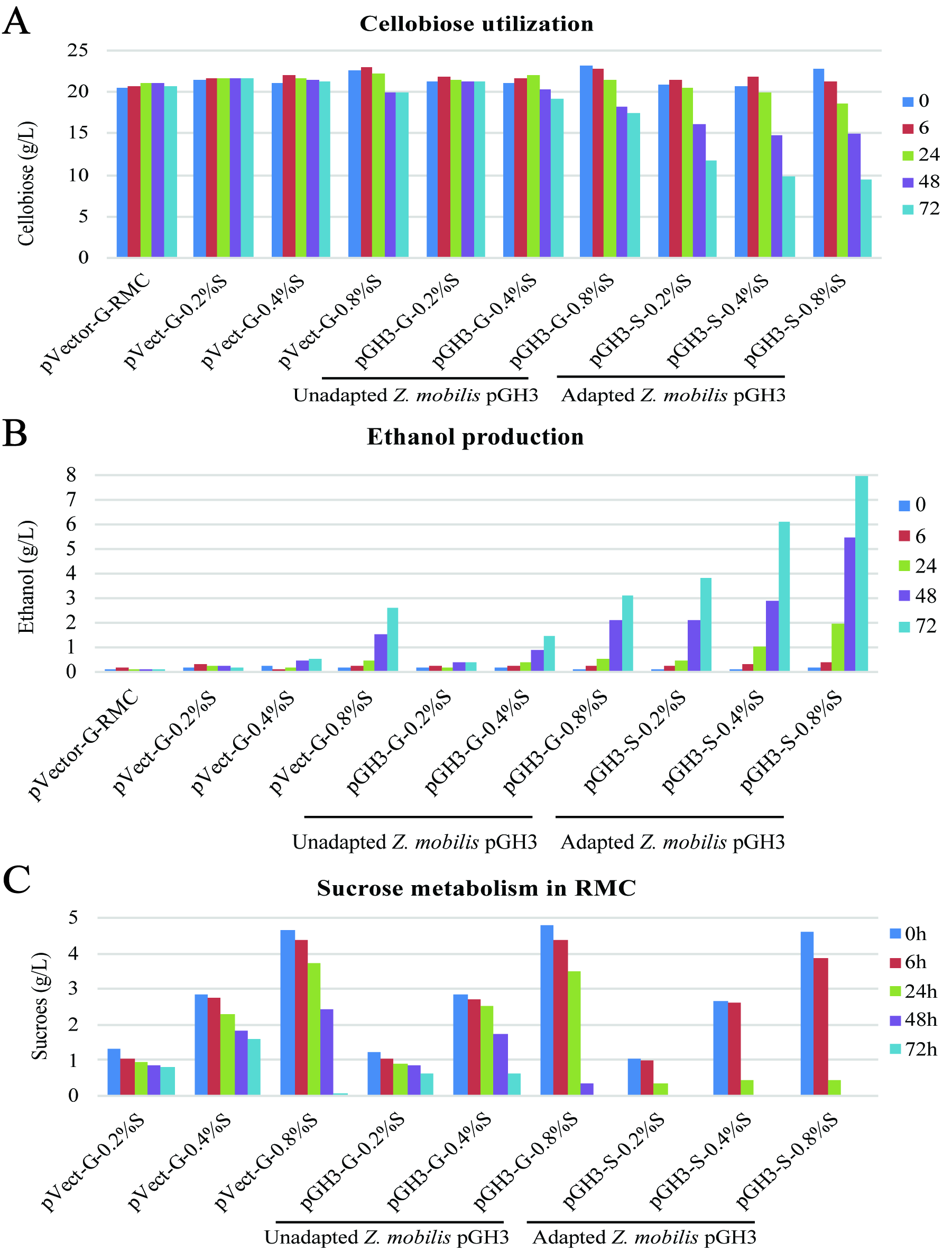

Supplement: S6 Fig — Cellobiose conversion (A), ethanol production (B) and sucrose metabolism (C) by unadapted Z. mobilis GH3, pVector control, and adapted Z. mobilis GH3 in an RMC medium with increasing concentrations of sucrose (0.2–0.8%). Z. mobilis containing pVector and pGH3 (pVect-G and pGH3-G, respectively) were pregrown in RMG medium. Similarly, Z. mobilis containing pVector and pGH3 (pVect-S and pGH3-S, respectively) were pregrown in RMS medium or increasing concentrations of sucrose (0.2%S– 0.8%S) in RMC. Samples were assayed at 0, 6, 24, 48, and 72 h (legend on right). (TIF) [file pone.0226235.s010.tif]

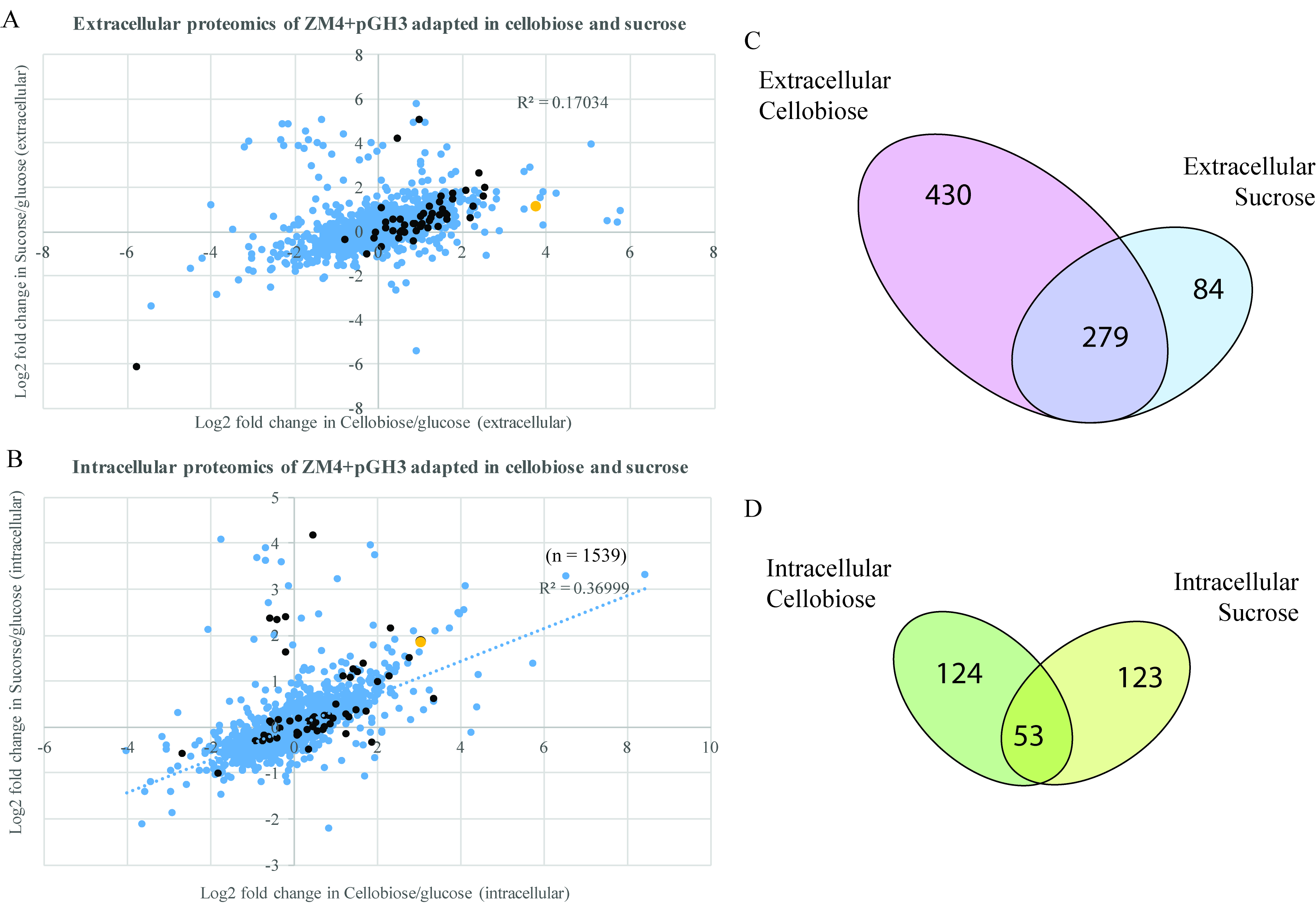

Supplement: S7 Fig — (A) Scatter plot of extracellular proteins of adapted strains grown on cellobiose or sucrose versus glucose, and (B) scatter plot of intracellular proteins of adapted strains grown on cellobiose or sucrose versus glucose. Proteins of interest are highlighted. Black–proteins related to secretion and transport, Orange–glycosyl hydrolase (CC_0968). (C) and (D) Venn diagrams showing overlap between changes in proteins for cells grown on cellobiose or sucrose versus glucose (adjusted p-value <0.001). (TIF) [file pone.0226235.s011.tif]

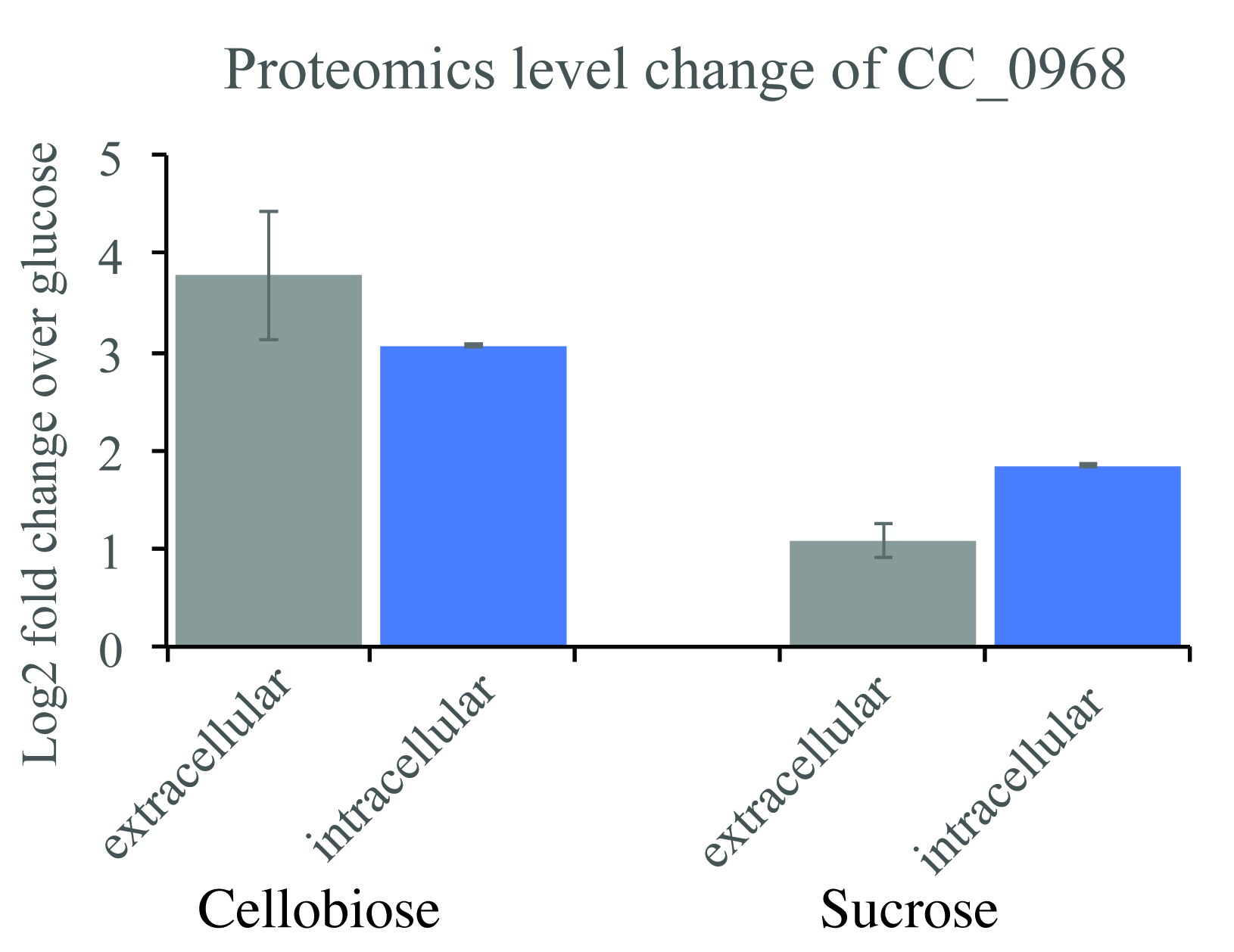

Supplement: S8 Fig — (TIF) [file pone.0226235.s012.tif]
